# Biosoftening of banana pseudostem fiber using cellulase and pectinase enzyme isolated from *Aspergillus niger* for textile industry

**DOI:** 10.1186/s43141-023-00617-3

**Published:** 2023-12-18

**Authors:** Manjusha W. A., Josphine J. S., Deepa P. K., Sujatha S., Ahil Raj S.

**Affiliations:** Department of Biotechnology, Malankara Catholic College, Kaliakkavilai, Tamil Nadu India

**Keywords:** Biosoftening, Cellulase, Pectinase, *Aspergillus niger*, Pseudostem fiber, SEM

## Abstract

**Background:**

Nowadays, farmers are facing a lot of problems for the disposal of banana pseudostem waste after the harvesting of banana. Banana pseudostem is a rich source of fiber, which is an alternative source of other natural and synthetic fibers. The banana fibers are biodegradable, and they are expected to be in great demand in the international market. For the textile industry, fibers were extracted using chemical and mechanical methods, but it leads to damage and affects the quality of fibers. So, this study mainly focused on biosoftening of banana pseudostem fiber using crude enzyme produced from *Aspergillus niger* which is one of the most predominant fungus which can synthesize industrially applicable enzymes and which can soften the surface of banana pseudostem fiber. Through this, biosoftened banana pseudostem fiber can be produced, and the disposal problem of banana pseudostem can be rectified in an eco-friendly manner.

**Result:**

The present study was undertaken for the biosoftening of banana pseudostem fiber using crude enzymes isolated from fungal strain. The fungal isolates were subjected to enzyme screening such as cellulase, pectinase, chitinase, peroxidase, and polygalacturonase. The maximum production of enzyme was observed in F2 strain, and it was subjected to crude enzyme production and purification using dialysis and column chromatography. The collected best enzyme fractions were selected for the biosoftening of banana pseudostem fiber. The banana pseudostem fiber was treated with crude enzymes at the time duration of 2, 4, 24, and 48 h. After the treatment, the treated and untreated fibers were evaluated for the mechanical properties and chemical constituent’s analysis. The results revealed that the chemical contents were high during 2- and 4-h-treated fibers. After that, chemical constituents were reduced due to the removal of debris by the action of enzymes. The mechanical properties such as breaking load, breaking extension, tenacity, and diameter of fiber were best in the fibers treated for 2 and 4 h. After 4 h due to the removal of chemical constituents the breaking load and tenacity, diameter will be reduced. SEM results proved that the fiber treated at 4th h showed smooth and softened fiber.

**Conclusion:**

This study proved that the crude enzymes isolated from the *Aspergillus niger* can be effectively soften and increase the quality of banana pseudostem fiber.

## Introduction

India is the largest producer of banana contributing to 27% of the world production [13]. The pseudostem is the fiber yielding part of banana plant. More than 50 million tonnes of banana pseudostem is generated after harvest of banana plant from which 3.9 million tonnes of fiber could be extracted [5]. Banana pseudostem fiber is a lignocellulosic material mainly consists of polysaccharides with cellulose microfibrils (50–60%) embedded with hemicelluloses (25–30%), lignin (12–18%), pectin (3–5%), water-soluble material (2–3%), wax and fat (3–5%), and ash (1–1.5%) [14]. Banana fiber exhibits better reinforcing efficiency and have good durability, strength, and resistance to environmental factors, and they are gaining importance in the international markets as they pose no toxic effects to mankind and the environment.

Currently, millions tonnes of banana pseudostem are dumped in our country as waste, and most of the farmers are facing problems in disposing it. This dumped banana pseudostem waste also causes environment hazards and makes ecosystem imbalance. Therefore, an effective economic means of reducing this environmental problem is needed in our country. To reduce this problem, it has to be planned to utilize the banana pseudostem waste in an economic and useful way. In view of this, it is planned to biosoften the banana fiber using enzymes produced from *Aspergillus niger*. The softened fiber is a good source of raw material in paper and textile industry. *Aspergillus* sp. is the most predominant used filamentous fungus that can synthesize various industrial enzymes such as cellulases, β-glucosidases, hemicellulases, pectinase, laccases, lipases, proteases, β-galactosidases, tannases, keratinase, cutinases, and aryl alcohol oxidase applicable in various industrial applications including pulp and paper, laundry, food, animal feed, brewery and wine, textile, and bioenergy industry [20]. The softened banana fibers also have lot of applications in industrial level. For the softening of banana pseudostem fiber mechanical, chemical, and biological treatments are in practice. When compared with other treatments, biological treatment using microbial enzymes showed better yield and quality of fiber. *Aspergillus niger* is a vigorous producer of softening enzymes, namely pectinase, cellulase, and laccase [22].

## Methods

### Isolation of fungal strain

The fungal isolates were isolated from decomposed banana litter collected from the cultivator site in clean plastic bags and transported to the laboratory in aseptic conditions. The collected (1 g) sample was serially diluted, plated in sterilized potato dextrose agar (PDA), and incubated at room temperature in dark conditions for 3–5 days. Morphologically, different colonies were subcultured and plated subsequently to obtain pure culture [16].

### Presumptive screening and determination of enzyme activity

The F1, F2, F3, and F4 fungus were individually screened for enzyme activity using carboxymethyl cellulose (CMC) plate assay [31] for cellulase, pectinase screening agar medium (PSAM) [3] for pectinase, and chitin agar plating [8] for chitin, and peroxidase and polygalacturonase activity was screened using the methods described by Lopez et al. [11] and Silva et al. [26], respectively. The crude extracts obtained from F1, F2, F3, and F4 were assayed for cellulase [7], pectinase, chitinase [28], peroxidase, and polygalacturonase [26] to check the maximum enzyme production. The high enzyme-producing strain was subjected to further studies.

### Crude enzyme production from fungal strain (F2)

#### Extraction of crude enzyme

One milliliter of 48-h-old culture of F1, F2, F3, and F4 (*Aspergillus niger*) was transferred into 100 ml of sterile mineral medium containing 0.5% citrus pectin and 0.5% lignin and incubated in the rotary shaker (150 rpm) at 30 °C for 5 days. The enzyme extract was separated by filtration followed by centrifugation at 10,000 rpm for 10 min and stored at 4 °C until use [6].

### Purification of crude enzyme from fungal (F2)

The crude extract was purified by 80% ammonium sulfate precipitation based on Ibraheem et al. [6]. The partially purified extract was subjected to dialysis using ice-cold 0.05-M citrate buffer (pH 4.0) for 24 h with continuous stirring using a magnetic stirrer and intermittent replacement of the buffer at 3–4-h intervals. The dialyzed fraction was packed on a column with CM-cellulose gel (2.5 × 30 cm, bead size 60–140 µ). The fractions were eluted with mobile phase (0.05-M citrate buffer (pH 4.0)). A flow rate of 0.1 ml/2 min was maintained, and the fractions were collected. The purified elutes were assayed for cellulase and pectinase, and highly active elutes were subjected to biosoftening of banana pseudostem fiber.

### Identification of fungal strain (F2)

The isolated fungus such as F1, F2, F3, and F4 was primarily examined for growth characteristics and biochemical characterization such as indole; methyl red; Voges-Proskauer and citrate utilization test (IMViC); sulfate, indole, and motility test (SIM); and catalase using standard protocol of Willey et al. [30]. The highly active fungal strain was subjected to molecular identification using 18S rRNA gene sequencing, and the sequence has been submitted to the GenBank database of the NCBI, and the phylogenetic tree was generated using Kimura 2-parameter model [9].

### Pseudostem fiber extraction

Banana pseudostems were collected from cultivation fields from Kanyakumari Dist. The stems were cut into small pieces of 1 m long and brought to the laboratory for fiber extraction. In the laboratory, samples were first surface sterilized with 5% sodium hypochlorate for 2 min, washed in sterilized water, and then air-dried for 2 h. The banana fiber can be extracted manually by cutting it into pieces of about 30 cm in length and 5 cm in width. Then the pseudostem was scraped, and the fiber was separated by using a flat blunt knife. Extracted fibers were washed thoroughly with clean tap water to remove the attached debris and gummy substances of pseudostem [17].

### Treatment of fibers using crude enzyme/partially purified enzyme elutes

Raw fibers of 1 g was taken in sterile Petri plates; to this, 10 ml of partially purified enzyme extract was added and subjected to treatment for 2 h (T1), 4 h (T2), 24 h (T3), and 48 h (T4), respectively. After treatment, the fibers were taken out and slowly washed under tap water. The fiber was allowed to air dry on filter paper. The enzyme-treated and untreated fibers were analyzed for microscopic characterization under a phase-contrast microscope (Nikon TE 2000) to evaluate the surface smoothness and evenness of the fiber.

### Analysis of mechanical and chemical properties of manually extracted banana pseudostem fiber

#### Mechanical properties—measurement of single fiber

Tensile strength was carried out using universal Instron tester, and breaking load and breaking extension were measured based on load developed and extensions at the point of rupture using computer software (Bluehill). The tenacity was calculated based on the breaking force and the specimen’s linear density, and the diameter of the individual fiber was calculated using ocular micrometry [18].

#### Chemical properties

The chemical constituents of the extracted fiber such as cellulose, hemicellulose, and pectin were estimated according to the method described by Sadasivam and Manickam [21], and lignin was estimated based on Thimmaiah’s method [27], and the chemical constituents were expressed in percentage.

### SEM analysis

The morphology of the biosoftened banana pseudostem fiber reinforced with crude extracts of *Aspergillus niger* interface was examined using a scanning electron microscope (SEM).

### Statistical analysis

The experiments were performed in triplicates, and the obtained results were statistically analyzed using MS Excel.

## Results

The present study mainly focuses on the biosoftening of banana psuedostem fiber using crude enzyme purified from *Aspergillus niger*. The enzyme extracted from F1, F2, F3, and F4 fungal isolates was subjected to cellulase, pectinase, chitinase, peroxidase, and polygalacturonase enzyme assay. According to an enzyme assay screening study, the fungal strain F2 showed maximum enzyme activity for cellulase, pectinase, chitinase, peroxidase, and polygalacturonase in-turns of 152.14, 149.02, 154.05, 121.25, and 109.19 (unit/ml), respectively. So, the F2 strain was selected for further enzyme production and biosoftening studies. The results were recorded in the Table 1.

**Table 1:** Quantification of enzyme present in crude extracts of fungal isolates

**Sl. no**	**Crude extracts of fungal isolates**	**Cellulase (unit/ml)**	**Pectinase (unit/ml)**	**Chitinase (unit/ml)**	**Peroxidase (unit/ml)**	**Polygalacturonase (unit/ml)**
1	F1	147.49 ± 0.01	127.22 ± 0.01	119.19 ± 0.02	111.03 ± 0.01	97.26 ± 0.01
2	F2	152.12 ± 0.01	149.06 ± 0.02	154.07 ± 0.02	121.26 ± 0.02	109.22 ± 0.03
3	F3	103.03 ± 0.01	89.22 ± 0.02	94.22 ± 0.01	81.57 ± 0.01	75.09 ± 0.02
4	F4	127.19 ± 0.02	98.29 ± 0.03	108.04 ± 0.02	94.06 ± 0.01	83.21 ± 0.01

### Crude enzyme production and purification

The high enzyme-producing F2 strain was subjected to crude enzyme production. Then, the culture filtrate was filtered and centrifuged, and the obtained crude extract was purified using ammonium precipitation and subjected to dialysis. The partially purified extract was eluted using column chromatography. The collected elutes were analyzed for enzyme activity. Elutes 3 and 4 showed maximum cellulase activity, and elutes 5 and 6 showed maximum pectinase activity. Elutes 3 and 4 (A) and elutes 5 and 6 (B) were mixed for the biosoftening of banana pseudostem. The results were recorded in Table 2.

**Table 2:** Purified column elutes of crude extract of F2

**Sl. no**	**Purified column elute**	**Cellulase (unit/ml)**	**Pectinase (unit/ml)**
1	E1	204.13 ± 0.01	10.7 ± 0.05
2	E2	253.8 ± 0.15	12.7 ± 0.05
3	E3	356.4 ± 0.2	21.4 ± 0.1
4	E4	329.5 ± 0.1	38.34 ± 0.01
5	E5	318.7 ± 0.17	70.2 ± 0.15
6	E6	304.01 ± 0.01	52.8 ± 0.15
7	E7	251.62 ± 0.01	22.1 ± 0.01
8	E8	198.57 ± 0.01	21.6 ± 0.05

### Identification of fungal strain (F2) by 18S rRNA sequencing

**Fig. 1:**
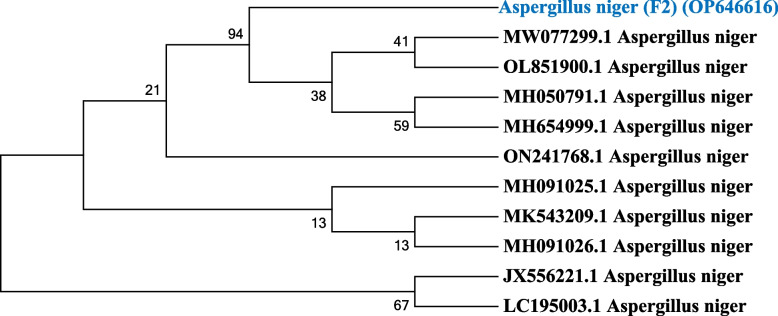
Phylogenetic tree analysis of the isolated strain (F2) (OP646616)

The isolated fungal strain (F2) was identified as *Aspergillus niger* by 18S rRNA gene sequencing, and the obtained sequence data has been submitted to the GenBank database of the NCBI with the accession number of OP646616. The identified fungal strain (F2) showed high similarity with *Aspergillus niger* based on nucleotide homology and phylogenetic analysis. The phylogenetic tree analysis of *Aspergillus niger* is shown in Fig. 1.

### Biosoftening of banana pseudostem fiber

Banana pseudostem fiber of 1 g was treated with 2.5 ml each of A and B and 5 ml of distilled water, subjected to biosoftening at the time interval of 2, 4, 24, and 48 h. The treated fibers were evaluated for its mechanical and chemical properties, and the results were tabulated in Tables 3 and 4. The tensile strength of untreated fiber was 26.29, and high tensile strength was noted as 26.01 (g/tex) on 4th hour of enzyme treatment. The breaking load was noted as 315.41 g for untreated fiber, and high breaking load was found as 313.44 g at the 4th h of treatment. The breaking extension of untreated fiber was 1.88% and showed maximum extension of 1.47% at 4th h of enzyme treatment. The tenacity of 12.05 (g/tex) was found in untreated fiber, and high tenacity of 11.11 (g/tex) was observed at 4th h of treatment. The diameter of untreated fiber was found to be 0.13 mm, and the treated fiber (4th h) showed 0.13 mm of diameter.

**Table 3:** Physical properties of pseudostem fiber extracted from Nendran banana

**Sl. no**	**Property**	**Untreated fiber**	**Enzyme-treated fiber**			
			**2 h**	**4 h**	**24 h**	**48 h**
1	Tensile strength (g/tex)	26.29 ± 0.04	26.12 ± 0.02	26.01 ± 0.04	23.12 ± 0.01	20.14 ± 0.03
2	Breaking load (g)	315.41 ± 0.02	314.05 ± 0.02	313.44 ± 0.02	310.07 ± 0.02	308.23 ± 0.02
3	Breaking extension (%)	1.88 ± 0.03	1.68 ± 0.01	1.47 ± 0.02	0.95 ± 0.01	0.85 ± 0.01
4	Tenacity (g/tex)	12.05 ± 0.02	11.88 ± 0.02	11.11 ± 0.01	10.71 ± 0.05	9.92 ± 0.03
5	Diameter (mm)	0.13 ± 0.01	0.13 ± 0.01	0.13 ± 0.01	0.12 ± 0.01	0.12 ± 0.01

**Table 4:** Chemical properties of pseudostem fiber extracted from Nendran banana

**Sl. no**	**Content**	**Untreated fiber (%)**	**Enzyme-treated fiber**			
			**2 h**	**4 h**	**24 h**	**48 h**
1	Cellulose	61.23 ± 0.02	60.24 ± 0.01	59.65 ± 0.01	57.15 ± 0.01	55.26 ± 0.01
2	Hemicellulose	16.76 ± 0.02	16.03 ± 0.02	15.47 ± 0.01	14.54 ± 0.02	13.22 ± 0.01
3	Pectin	1.56 ± 0.01	1.52 ± 0.01	1.46 ± 0.01	1.23 ± 0.02	1.17 ± 0.02
4	Lignin	15.75 ± 0.02	14.04 ± 0.01	13.54 ± 0.01	10.06 ± 0.01	9.52 ± 0.01

Normally, the cellulose content of the banana pseudostem fiber varies in the range of 55.26 to 61.23%. In the present study, the untreated fiber contains 61.23%, and the treated fiber (4th h) showed 59.65% of cellulose. The hemicelluloses, pectin, and lignin content of untreated fiber was 16.76, 1.56, and 15.75%, respectively. Among the enzyme-treated fibers, 4-h treated fiber showed hemicelluloses, pectin, and lignin content of 15.47, 1.46, and 13.54%, respectively. The reduction of chemical content indicates the removal of debris by the activity of enzyme.

### SEM analysis

**Fig. 2:**
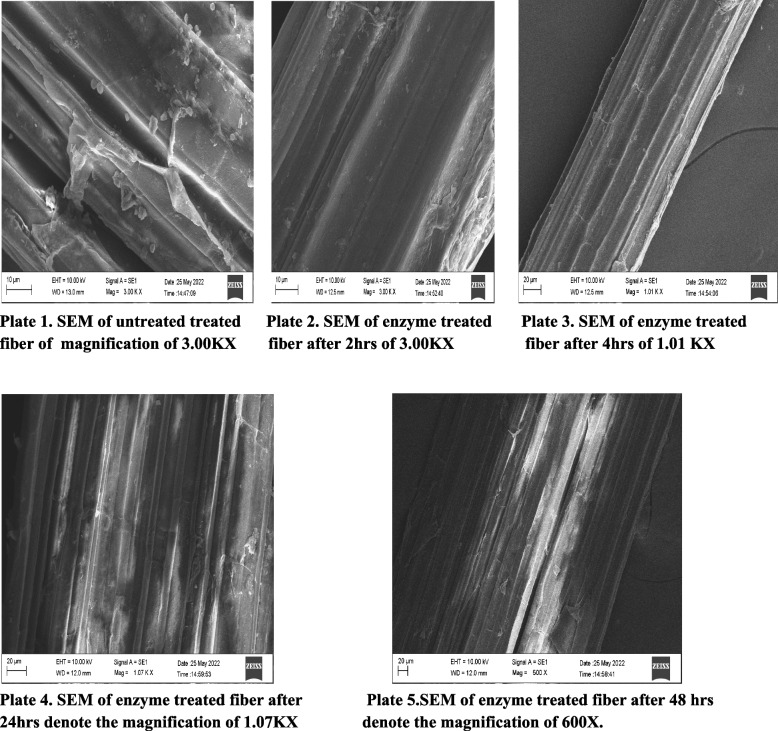
SEM images of untreated and enzyme-treated banana pseudostem fibers. Plate 1 SEM of enzyme untreated fiber. Plate 2 SEM of enzyme treated fiber after 2 hrs. Plate 3 SEM of enzyme treated fiber after 4 hrs. Plate 4 SEM of enzyme treated fiber after 24 hrs. Plate 5 SEM of enzyme treated fiber after 48 hrs

SEM analysis confirms the better removal of chemical contents such as lignin, pectin, cellulose, and hemicellulose, without damaging the fibrils, which results increasing in tenacity. It indicates the binding materials on the surface of untreated fibers which showed rough and irregular surface. In the treated fiber, 4–48 h showed clear smooth surface which indicates the removal of binding materials, due to the action of enzyme such as cellulase and pectinase. The treated and untreated fiber SEM images are shown in Fig. 2.

## Discussion

Currently, dry banana fibers are used from the pseudostem for industrial applications. For the separation of fiber from the banana pseudostem, mechanical extraction is normally practiced, due to its higher efficiency. But that fiber showed high rough surface and more unevenness due to the non-removal of chemical constituents. Before composite preparation/spinning, the extracted fibers were degummed through softening using chemical or enzymes. Normally, cellulase and pectinase are used for the softening of fiber. The present study mainly focuses on the evaluation of softening of banana fiber using crude enzymes isolated from *Aspergillus niger*. Sattar et al. [23] demonstrated that *Aspergillus* sp. could produce enzymes for industrial applications.

Cellulose is one of the major components of the pseudostem fiber. In the present study, the cellulose content of the banana pseudostem fiber varies in the range of 55.26 to 61.23%. The untreated fiber contains 61.23%, and the treated fiber (4th h) showed 59.65% of cellulose. The hemicelluloses, pectin, and lignin content of untreated fiber were 16.76, 1.56, and 15.75%, respectively. Among the enzyme-treated fibers, 4-h-treated fiber showed maximum hemicelluloses, pectin, and lignin content of 15.47, 1.46, and 13.54%, respectively. Preethi and Balakrishna [18] studied the physical and chemical properties of banana pseudostem, and peduncle fibers collected from Grand Naine, Poovan, Monthan, and Nendran among the highest cellulose content of 59.22% were noted in pseudostem fiber of Nendran, and the other noncellulosic content was high in Monthan pseudostem fiber and in Nendran 12.09, 14.39, and 2.68% of hemicellulose, lignin, and pectin, respectively. According to Amrutha et al. [2], the cellulose content was found to be 60.43% in Nendran than that of Karpuravalli 66.56%, and high percent of hemicellulose and lignin content was observed in Nendran fibers at 15.90% and 17.11%, respectively. The mean breaking strength and elongation and tenacity of Nendran were found to be 4.61 N, 2.15%, and 5.23 g/tex, respectively, for the banana fibers collected from Karpuravalli, Nendran, Robusta, red banana, Palayamkodan, and Neypoovan.

After ripe banana fruits are harvested, banana stems are processed, primarily by cutting them and transporting them to the field for free stacking. They may also be discarded on the spot and left to rot, thereby taking up land resources, causing environmental pollution, and wasting resources [1]. According to Li et al. [10], the fibers obtained from banana pseudostem have good elasticity, tensile strength, and stiffness capacity. The physical properties include the diameter of 0.119 mm, 3.21 N of breaking force, 288.7 MPa of tensile strength, and 1.67% of fiber extension. Shivashankar et al. [24] reported that fibers extracted from Nendran contain 60% cellulose, and 20% lignin and strength characteristics such as mean breaking load and extension and tenacity were comparably high than that of naturally occurring plant fibers such as pineapple, jute, and sisal. Higher cellulose content contributes to the higher mechanical strength of fiber, which makes it preferable for textile, paper, and other applications and the hemicelluloses link the cellulose microfibrils together and upon degradation result in lower fiber bundle strength. Lignin acts like a matrix material within the fibers, making stress transfer, and plays a crucial role in conducting water in plant stem. Mechanical properties such as texture and fiber diameter decide the fineness. The tensile strength of untreated fiber was 26.29, and high tensile strength was noted as 26.84 (g/tex) on 4th hour of enzyme treatment. The breaking load was noted as 315.41 g for untreated fiber, and high breaking load was found as 313.44 g at the 4th h of treatment. The breaking extension of untreated fiber was 1.88% and showed maximum extension of 1.47% at 4th h of enzyme treatment. The tenacity of 12.05 (g/tex) was found in untreated fiber, and high tenacity of 11.91 (g/tex) was observed at 4th h of treatment. The diameter of untreated fiber was found to be 0.13 mm, and the treated fiber (4th h) showed 0.13 mm of diameter. The impurity content of fiber extracted by the chemical method is low, but the tensile strength and toughness of this fiber seriously decline [4].

Preethi and Balakrishna [18] reported that fine fiber strength was noted in the pseudostem fiber of Nedran with a texture of 24.23 and 0.119 mm in diameter. Banana pseudostem fibers extracted mechanically have shown to be of unevenness and rough surface due to the presence of non-removable biomolecular constituents such as pectin, lignin, and hemicelluloses, and the extracted fibers are subjected to spinning to free the gum residues for composite preparation. Since mechanical process is a tedious chemical or enzymatic treatment is preferred. Commonly used enzymes for the softening of fibers include pectinase, cellulase, and laccase. Those enzymes are extracted from the fungal strain *Aspergillus niger* [22]. Similarly, Vellaichamy and Gaonkar [29] evaluated the softening of mechanically extracted banana pseudostem fiber using the fungal isolates *Aspergillus niger*. The mechanically extracted fibers are subjected to crude enzyme treatment at different time duration from 2 to 168 h, and the mechanical and chemical constituents are evaluated for treated and untreated fibers. According to the current study, the fibers treated with crude extract of 4 h showed better mechanical and softness properties than other hour treatments.

Scanning electron microscope (SEM) provides an excellent exposition of the surface morphology of banana fibers. Reddy and Yang [19] stated that the banana fibers extracted physically contain an irregular surface with the presence of encrusting substances like hemicellulose, lignin, and pectin. The main quality parameter of raw textile material is fiber length because length-to-width ratio is the primary requirement of any textile fiber. It is clear from SEM images of the banana fibers that they contain bundles of individual cells that have been bounded together by lignin. Manilal and Sony [12] reported that scanning electron microscopy (SEM) studies showed clean and smooth surfaces of bio-extracted fibers, unlike physically extracted ones. Mumthas et al. [15] proved that enzymatically extracted and softened banana fiber showed higher tenacity and strength values than that of chemical and mechanical extraction method. Shroff et al. [25] stated that combination of more than two enzymes such as hemicellulase, pectinase, and cellulase showed better soften and impurities removal on the surface of banana pseudostem fiber than that of single enzyme treatment.

In the present study, the untreated fibers showed rough and irregular surface due to the deposition of adhesives. The fibers treated for 4 h showed better clear and smooth surface than 2-h-treated fiber. The results indicate that the softening of fibers is mainly due to the removal of adhesives by the action of enzymes such as cellulase and pectinase. After 4 h, the excess removal of chemical constituents by the action of enzyme leads to the loss of tensile strength, tenacity, and diameter. The results suggest the biosoftening of banana fiber using cellulase and pectinase enzyme isolated from *Aspergillus niger* is a best way to produce softened fiber in textile industry.

## Conclusion

In the present study, the biosoftening of banana pseudostem fiber using enzymes such as cellulase and pectinase isolated from *Aspergillus niger* was evaluated. The banana pseudostem fiber was subjected to enzyme treatment from *Aspergillus niger* to improve its biosoftening and mechanical properties. The results showed that the fibers treated with cellulase pectinase enzyme of 4 h had better mechanical and softness properties, while the untreated fibers showed rough and irregular surface due to the deposition of adhesives. In the other treated fibers, the excess removal of chemical constituents by the action of enzyme leads to the loss of tensile strength, tenacity, and diameter. The results indicate that the softening of fibers is mainly due to the removal of adhesives by the action of cellulase and pectinase enzyme, and it was confirmed by SEM. The present study concluded that biosoftening of banana fiber using enzyme isolated from *Aspergillus niger* could be an ideal treatment for the banana pseudostem fiber in textile industry, and the banana pseudostem waste can be reused in an eco-friendly manner.

## Data Availability

Data and material will be provided on the request.
